# A systematic review of the willingness-to-accept and willingness-to-pay disparities in empirical studies in the healthcare field

**DOI:** 10.1186/s13690-025-01699-w

**Published:** 2025-08-18

**Authors:** Yue Wang, Xiaoyu Yan, Hongchao Li

**Affiliations:** 1 School of International Pharmaceutical Business, China Pharmaceutical University, Nanjing, Jiangsu China; 2 School of International Pharmaceutical Business and Center for Pharmacoeconomics and Outcomes Research, China Pharmaceutical University, Nanjing, Jiangsu China

**Keywords:** Healthcare field, Willingness-to-accept, Disparity, Cost-effectiveness threshold

## Abstract

**Purpose:**

Willingness-to-pay (WTP) and willingness-to-accept (WTA) are widely used measures of individual preferences in valuing healthcare services; however, a persistent disparity between them, often with WTA exceeding WTP, raises concerns. This study aims to review empirical research evidence to achieve a comprehensive understanding of the disparity between WTA and WTP for health outcomes or healthcare goods and services.

**Methods:**

A search was conducted in PubMed, Embase, Web of Science, and Scopus from inception to November 15, 2023, for empirical research articles reporting both WTA and WTP in the healthcare field. Data extracted from the included studies encompassed WTA and WTP values, participation response rates, and other study characteristics. Descriptive analyses were conducted to compare WTA/WTP ratios across studies, and chi-square tests were applied to examine differences in response rates where applicable.

**Results:**

A total of 779 records were identified through database searches. After removing duplicates, 405 records remained for title and abstract screening. Of these, 70 articles were retrieved for full-text review, and 28 articles met the eligibility criteria for inclusion in the final qualitative analysis, encompassing 35 distinct studies or subgroups. The reported WTA/WTP ratios ranged from 0.14 to 29.19, with a median value of 1.61, indicating that individuals often demand higher compensation to give up healthcare benefits than they are willing to pay to obtain them. Among the empirical studies analyzed, 29 studies (82.86%) from 24 articles reported WTA values that exceeded WTP values, while 6 studies (17.14%) from the remaining 4 articles indicated WTA values lower than WTP values. Among the 14 studies reporting both WTA and WTP response rates, six studies indicated a significantly lower WTA response rate compared to the WTP response rate, whereas two studies found the WTA response rate to be significantly higher (*P* < 0.05). The WTP response rate was observed to range from 0.89 to 20.23 times that of the WTA response rate.

**Conclusions:**

The results of this study suggest that losses in health outcomes or healthcare goods and services are valued differently than gains. The disparities between WTA and WTP are influenced by various factors, including the income effect and personal preferences. Individual preferences shape perceptions of WTA and WTP questions, resulting in varied response rates. Considering these disparities in the medical and healthcare fields can assist policymakers in making more informed decisions regarding the allocation of medical and health resources.

**Supplementary Information:**

The online version contains supplementary material available at 10.1186/s13690-025-01699-w.

**Table:** 

Text box 1. Contributions to the literature	
The values of WTA and WTP vary across empirical studies concerning different healthcare goods, with a general trend of WTA being higher than WTP. The disparity between WTA and WTP may shift the cost-effectiveness threshold, meaning that healthcare interventions which are less effective but less costly may need stronger justification to be considered cost-effective. Decisions involving interventions that are less effective but less costly, typically represented in the lower-left region of the cost-effectiveness plane, deserve more attention due to the challenges in determining their value and the implications for resource allocation.	

## Introduction

In the medical and health systems, the prices individuals pay for goods and services in the medical market do not necessarily reflect the true value of these goods and services to patients. This discrepancy arises from several issues, including asymmetric information between doctors and patients, imbalances in supply and demand, and the influence of third-party payment mechanisms [1]. To better assess healthcare preferences and capture the value of health goods and services, health economists often employ contingent valuation methods (CVM). CVM is a stated preference technique used to assess the monetary value individuals assign to non-market goods, such as health or environmental benefits [2]. Unlike revealed preference methods, which infer value based on observed behavior in actual markets (e.g., travel cost or purchasing decisions), stated preference methods rely on individuals’ responses to hypothetical scenarios presented in surveys. Grounded in the principle of utility maximization, CVM was originally developed in environmental economics and has since been widely used to value non-market goods, including those in healthcare [3].

In the healthcare sector, CVM typically elicits the monetary value of medical products or services through surveys based on hypothetical scenarios [4]. The common forms of CVM include willingness-to-accept (WTA) and willingness-to-pay (WTP). WTA measures the minimum compensation an individual would require to forgo a specific health outcome, medical product, or service, while WTP assesses the maximum amount an individual is willing to pay for a particular health outcome, product, or service.

A notable trend in published empirical studies is the substantial difference between WTA and WTP values for the same good or service, with WTA often exceeding WTP [5]. The WTA/WTP ratio can vary significantly with the value of the commodity. For instance, studies on environmental products have reported a maximum WTA/WTP ratio of 6.23 [6]. In the healthcare domain, the earliest review of WTA/WTP ratios dates back to 2002 and included only two studies reporting ratios of 1.9 and 6.4 [7]. A ratio greater than 1 indicates that individuals demand more compensation to give up a health benefit than they are willing to pay to acquire it (WTA > WTP), often reflecting loss aversion or an endowment effect [8]. In contrast, a ratio less than 1 implies that individuals are willing to pay more to obtain a benefit than they would accept to lose it (WTP > WTA), which may occur in certain public good contexts or when substitutes are readily available [9]. The most recent systematic review, published in 2020, encompassed 13 studies related to health goods and services [1], yet its search timeframe was limited to 2017, rendering it somewhat outdated and lacking comprehensive coverage of healthcare products and services. This highlights the necessity for updated research to yield more relevant and complete findings. Understanding the relationship between WTP and WTA is crucial because it can directly influence healthcare decision-making. If policymakers rely solely on WTP to define value, they may undervalue losses and unintentionally disadvantage existing patients. Conversely, using WTA may result in higher compensation thresholds that influence decisions about disinvestment or reimbursement withdrawal. Knowing which measure tends to dominate under different conditions helps health economists and decision-makers choose the appropriate valuation approach for different policy contexts, particularly when assessing trade-offs under resource constraints.

The existing literature presents various explanations for the discrepancies between WTA and WTP. According to standard economic theory, WTA and WTP should converge when commodity values are divisible and can be exchanged at zero transaction costs within an infinitely large market. However, if these conditions are not met, WTA and WTP may diverge. The magnitude of this difference is influenced by factors such as income, the proportion of income allocated to goods, and income elasticity [10, 11].

Understanding the WTA/WTP ratio for medical products and services is crucial, as the differences between WTA and WTP can significantly impact medical decision-making. Insights into the WTA/WTP ratio of healthcare products and services can enhance the understanding of health insurance decisions, particularly since decision-makers may find it more challenging to discontinue reimbursement for a medical intervention than to refrain from initiating it [12]. Additionally, the WTA/WTP disparity aids in quantifying the compensation required to address health losses or benefits arising from changes in specific healthcare services. In pharmacoeconomic evaluations, the observed disparity between WTA and WTP suggests that the threshold for judging whether a healthcare intervention is worthwhile may differ depending on whether the intervention is more effective and more costly, or less effective but less costly. In particular, interventions that save money but offer slightly reduced health benefits may require a higher bar for acceptance, due to the way people value losses versus gains [7]. Therefore, consideration of the WTA/WTP ratio can inform the selection of appropriate value measures for medical and health interventions in the context of existing decision-making frameworks, such as determining whether to continue reimbursement for an existing treatment, assessing value in disinvestment scenarios, or evaluating new technologies with marginal benefit but substantial cost savings.

The purpose of this study is to review the available research evidence regarding the WTA/WTP disparity for healthcare products and services. This review aims to provide a general estimate of the WTA/WTP ratio in the healthcare field, thereby fostering a comprehensive understanding of the WTA/WTP disparity.

## Methods

### Literature search strategy

A comprehensive systematic search was conducted in four electronic databases: PubMed, Embase, Web of Science, and Scopus, from inception to 15 November 2023. The search strategy employed a combination of keywords and subject terms related to “willingness-to-pay”, “willingness-to-accept” and “healthcare”, including variations in titles, abstracts, and keywords. The full search strategies for each database are detailed in Additional file 1.

### Eligibility criteria

Studies were included if they met the following criteria: (1) they were empirical studies that reported both WTA and WTP estimates for the same or comparable health goods, services, or outcomes; (2) they were published in English; and (3) full-text articles were available for screening and analysis. Studies available only as abstracts, conference proceedings, or citation records were excluded. To minimize the risk of omission due to database limitations, we also manually reviewed the reference lists of relevant systematic reviews on WTA/WTP disparities and included additional eligible empirical studies that were not captured through the initial database search.

### Study selection process

After removing duplicates, two reviewers independently screened the titles and abstracts of the retrieved records according to the eligibility criteria. If the abstract did not provide sufficient information to determine eligibility, the full text was retrieved for further assessment. Discrepancies between reviewers were resolved through discussion, and a third reviewer was consulted if consensus could not be reached. Full-text screening of all potentially eligible studies was performed by one primary reviewer, with input from the other reviewers in cases of uncertainty. The study selection process was presented in a PRISMA flow diagram, which outlines the number of records identified, screened, assessed for eligibility, and ultimately included in the qualitative synthesis.

### Data extraction

A standardized data extraction form was used to collect key information from each included study, including: WTA and WTP estimates (used to calculate the WTA/WTP ratio); study characteristics such as first author, year of publication, country of origin, and World Bank defined income level (low-, middle-, or high-income); the type of health good, service, or health outcome valued, categorized into four domains (e.g., informal care, medical products/services, outcomes with uncertainty or other); sample characteristics including sample size and respondent type (e.g., patients, patient’s guardians, caregiver or public); the elicitation method (e.g., interviews, online surveys, or face-to-face surveys); and the response rate, defined as the proportion of participants who provided valid responses to WTA or WTP valuation questions.

### Data synthesis and analysis

We used descriptive statistics to summarize the characteristics of the included studies and the calculated WTA/WTP ratios. Ratios were stratified and compared across different respondent types, income-level groups, and categories of health goods or services. Where available, chi-square tests were used to assess statistically significant differences in response rates between WTA and WTP estimates.

## Results

**Fig. 1:**
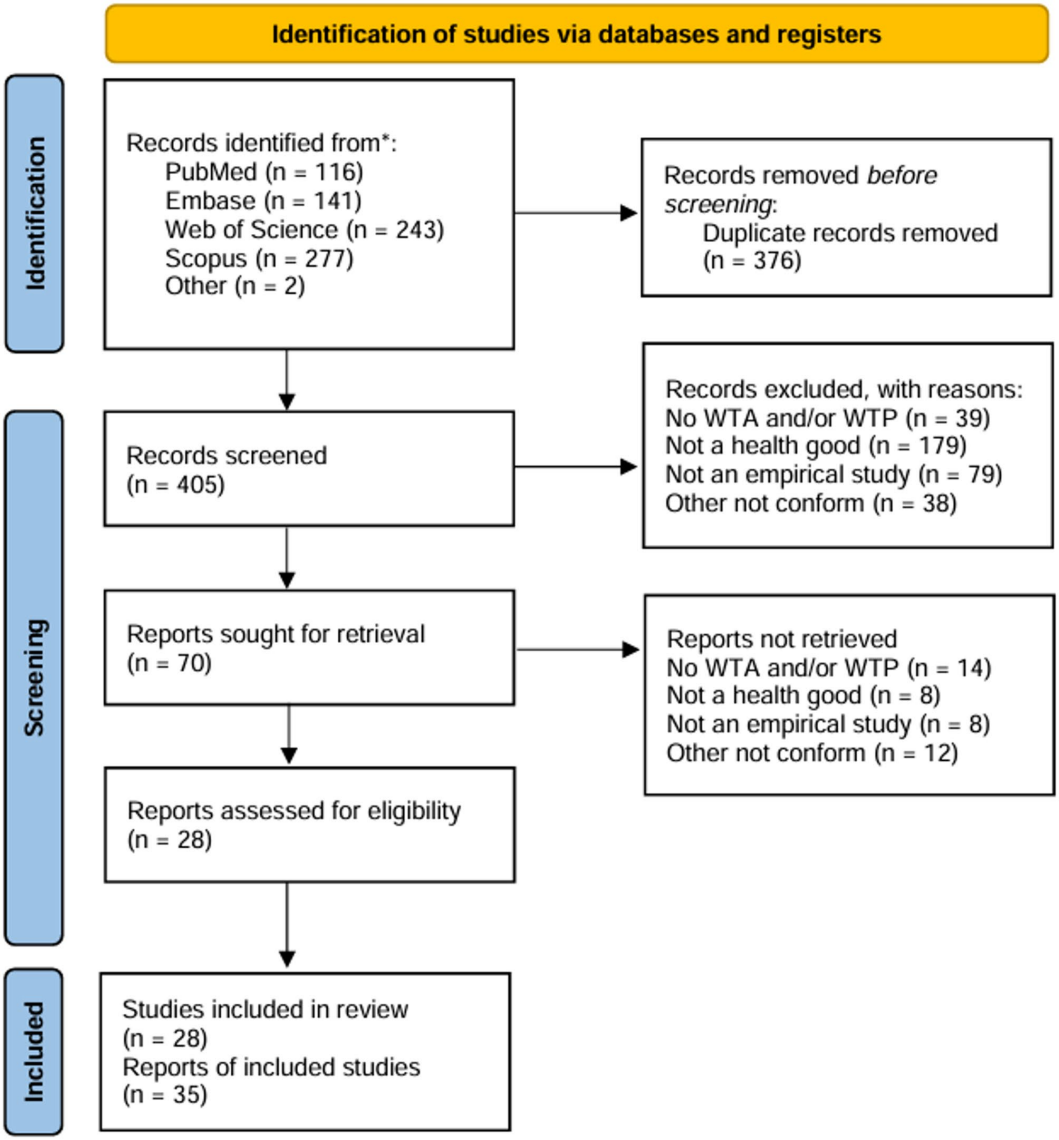
PRISMA flow diagram

Databases or platforms were searched on the 15 November, 2023. In total, 779 records were identified, and after the removal of duplicates, 405 remained for the title and abstract screening. We included 70 articles in full-text screening, and 28 stayed for qualitative analysis after the exclusion of 42 papers with reasons (for exclusion details, see Fig. 1).

### Descriptive characteristics of included studies

Table 1 presents the descriptive characteristics and the extracted WTA/WTP ratios from the studies included in this review. A total of 28 articles concerning WTP and WTA in the healthcare sector were analyzed [13–40]. These articles provided estimates for 35 studies or subgroups. The descriptive characteristics of the empirical studies, along with data related to WTP and WTA, were systematically extracted from the included articles, with detailed information summarized in Table 1. Statistical significance between the response rate of WTA and WTP was determined using the chi-square test. When *p* < 0.05, the differences between the two methods were deemed to be statistically significant.

**Table 1:** The included studies’ descriptive characteristics and the WTA/WTP ratio

Number	Author	Year	Nation	Income Level	Healthcare Goods/Service	*N*	Respondent	Intervention Methods	Response Rate			WTA/WTP ratio
									WTA	WTP	P	
1	Carthy [13]	1998	United Kingdom	High-Income	Non-fatal road injury£¨2 weeks hospitalization; full recovery after 18 months£©	167	Public	Survey	-		-	6.42
					Non-fatal road injury£¨2¨C3 days hospitalization; full recovery after 3¨C4 months£©	167	Public	Survey	-		-	6.9
2	O¡¯Brien [14]	1998	Canada	High-Income	Cancer drug Filgrastim£¨Prior Risk of Cancer 1/100£©	220	Public	Interview	-		-	2.34
					Cancer drug Filgrastim£¨Prior Risk of Cancer 1/200£©	220	Public	Interview	-		-	1.61
3	Tsuji [15]	2002	Japan	High-Income	Tele-health system	230	Patients	Survey, Interview	53.10%		-	3.6
4	Borisova [16]	2003	United States	High-Income	Methadone maintenance	303	Patients	Survey	66%		-	1.31
5	van den Berg [17]	2005	Netherlands	High-Income	Informal care	149	Patients	Survey, Interview	40.80%		-	1.05
						149	Care Providers	Survey, Interview	40.80%		-	1.22
6	Morey [18]	2007	United States	High-Income	Depression	104	Patients	Survey, Face-to-face Interview	-		-	1.09
7	Whynes [19]	2007	United Kingdom	High-Income	Pediatric cochlear implantation	216	Patient¡¯s guardians	Interview	31.50%	90.30%	<0.001*	4.01
8	Grutters [20]	2008	Netherlands	High-Income	Hearing aid	291	Patients	Survey, Interview	97.33%	96.60%	0.632	2.95
9	Mart¨ªn-Fern¨¢ndez [21]	2010	Spain	High-Income	Visit to the family physician	451	Patients	Interview	96.20%	92.50%	0.233	1.75
10	Meijer [22]	2010	Netherlands	High-Income	Informal care	787	Patients	Mail survey	31.90%	33.20%	0.591	1.3
						1453	Care Providers	Mail survey	57.80%	51.30%	<0.001*	1.15
11	Viscuis [23]	2012	United States	High-Income	Risk of gastrointestinal diseases caused by drinking water	4745	Public	Online survey	69%		-	2.09
12	Chilton [24]	2012	United Kingdom	High-Income	Health effect (Stomachache for one week)	156	Public	Survey	-		-	11.41
					Health effect (Throat ache for one week)	156	Public	Survey	-		-	9.84
13	Mart¨ªn-Fern¨¢ndez [25]	2013	Spain	High-Income	Primary care nursing consultation	662	Patients	Survey, Interview	95.90%	87.80%	<0.001*	1.34
14	Bayen [26]	2014	France	High-Income	Informal care	98	Patients	Scale	-		-	0.7
15	Chiwaula [27]	2016	Malawi	Low-Income	Informal care	93	Patients	Survey, Face-to-face Interview	-		-	2.4
16	Chiwaula [28]	2016	Malawi	Low-Income	Informal care	130	Care Providers	Survey, Face-to-face Interview	-		-	5.18
17	Finkelstein [29]	2016	Singapore	High-Income	Life-extending treatment at the end of life	290	Patients	Interview	-		-	0.77
					Quality-of-life-enhancing treatment at the end of life	290	Patients	Interview	-		-	0.77
18	Sendi [30]	2017	Switzerland	High-Income	Dental Implants	16	Patients	Survey, Telephone Interview	31.25%	93.75%	<0.001*	7.27
19	Gleason-Comstock [31]	2017	United States	High-Income	Time and travel costs to hospital	38	Patients	Survey, Face-to-face Interview	-		-	0.55
20	Oliva-Moreno [32]	2019	Spain	High-Income	Informal care	604	Care Providers	Survey, Interview	-		-	2.05
21	Isah [33]	2019	Nigeria	Middle-Income	Prevention of HIV Mother-to-Child Transmission Services	104	Patients	Survey	4.80%	97.10%	<0.001*	29.19*
22	Rezaei [34]	2020	Iran	Middle-Income	Vaccine	667	Patient¡¯s guardians	Survey	94.20%	93.10%	0.433	1.55
23	Liu [35]	2020	China	Middle-Income	Informal care	439	Care Providers	Face-to-face Interview	66.80%	73.90%	0.022*	1.53
24	Isah [36]	2021	Nigeria	Middle-Income	Prevention of HIV Mother-to-Child Transmission Services(UNTH)	120	Patients	Survey, Interview	17.50%	75.80%	<0.001*	0.14
					Prevention of HIV Mother-to-Child Transmission Services(ABUTH)	99	Patients	Survey, Interview	36.40%	61.60%	<0.001*	0.16
25	Carpio [37]	2021	United States	High-Income	Vaccine	1895	Public	Online survey	-		-	1.01
26	Mart¨ªn? Fern¨¢ndez [38]	2021	Netherlands	High-Income	Physician assistants replace anaesthesiologists	1905	Public	Online survey	94.75%	94.60%	0.828	3.55
27	Ramezani-Doroh [39]	2023	Iran	Middle-Income	Informal care	425	Care Providers	Survey, Telephone Interview	42.82%		-	1.54
28	Oliva [40]	2023	Spain	High-Income	Informal care	610	Care Providers	Survey	94%	92.60%	0.36	1.92

* denotes a significant difference between WTA and WTP response rates.

ABUTH: Ahmadu Bello University Teaching Hospital; UNTH: University of Nigeria Teaching Hospital

### Categories of health outcomes, goods and services

The application of CVM in the healthcare field primarily focuses on medical services and preventive measures, with informal care accounting for 31.43% of the research. Outcome uncertainty related to medical goods or services constitutes 25.71%, while other medical services account for 20%. In contrast, there is relatively less research on drug treatments (8.57%), vaccines (5.71%), and medical instruments (8.57%). Among these four categories, interventions involving outcome uncertainty had the highest median WTA/WTP ratio (3.55), followed by medical products or services (1.98) and informal care (1.53). The lowest median ratio was observed in other medical-related services (1.09), though this group showed the widest range (from 0.14 to 29.19). This suggests that uncertainty about health outcomes tends to amplify the divergence between individuals’ WTA and WTP. Table 2 presents the distribution of WTA/WTP ratios across four categories of health outcomes, goods and services.

**Table 2:** WTA/WTP ratio by four types of categories of health outcomes, goods and services

Categories of health outcomes, goods and services	Studies/subgroups (*N*)	Median value of WTA/WTP	WTA/WTP range
Informal care	11	1.53	0.7–5.18
Outcome uncertainty related to medical goods or services	9	3.55	0.77–11.41
Medical products or services	8	1.975	1.01–7.27
Other medical related services	7	1.09	0.14–29.19

### Respondent type

The respondents in the studies primarily included patients (48.57%), healthcare providers (20%), members of the general public (25.71%), and patient’s guardians. The WTA/WTP ratio varied across different respondent groups. On average, studies involving patients and caregivers tended to report lower WTA/WTP ratios (median values of 1.3 and 1.54, respectively), while those involving the general public or caregivers showed higher ratios. Detailed subgroup-level ratios by respondent type are provided in Table 3.

**Table 3:** WTA/WTP ratio by four types of respondents

Respondent type	Studies/subgroups(*N*)	Median value of WTA/WTP	WTA/WTP range
Patients	17	1.3	0.14–29.19
Patient’s guardians	2	2.78	1.55–4.01
Care Providers	7	1.54	1.15–5.18
Public	9	3.55	1.01–11.41

### Income level

When stratified by national income level, 27 studies/subgroups from high-income countries reported a median WTA/WTP ratio of 1.75. The included middle-income country studies reported a median ratio of 1.54. In contrast, studies from low-income countries showed a higher median ratio of 3.79. See Table 4 for detailed results by World Bank income classification.

**Table 4:** WTA/WTP ratios by world bank income groups

World Bank Income Groups	Studies/Subgroups (*N*)	Median value of WTA/WTP	WTA/WTP range
Low-income countries	2	3.79	2.4–5.18
Middle-income countries	6	1.54	0.14–29.19
High-income countries	27	1.75	0.55–11.41

### Response rate

A total of 28 articles were included in the analysis, encompassing 35 studies or subgroups related to WTA and WTP. Among these 35 studies, 20 reported the response rates of participants. Of these, 14 studies provided both WTA and WTP values, while the remaining six reported WTA values only. Among the 14 studies that reported both WTA and WTP values, eight demonstrated statistically significant differences in response rates between WTA and WTP (*P* < 0.05); in six of these, the response rate for WTA questions was significantly lower than that for WTP, while in two studies, the WTA response rate was higher. This suggests that participants were generally less likely to answer WTA-type questions compared to WTP-type questions. Overall, the WTP response rate was found to range from 0.89 to 20.23 times that of the WTA response rate.

### Summary of WTA/WTP ratios

The WTA/WTP ratios extracted from the included articles ranged from 0.14 to 29.19, with a median value of 1.61. There were substantial disparities in the average WTA/WTP ratios across different studies. Among the 35 experiments, 29 (82.86%) indicated higher WTA values than WTP values. The highest WTA/WTP ratio of 29.19 was observed in a study focused on the prevention of mother-to-child transmission (PMTCT) services [33]. The second highest was found in a study examining the health effects of stomachache and sore throat, which reported WTA/WTP values of 11.41 and 9.84, respectively [24]. The remaining 26 studies reported WTA/WTP values ranging from 1.05 to 7.27, exhibiting slight variations between the ratios, consistent with findings from previous reviews [1].

In addition to the common trend of higher WTA values compared to WTP values, cases were also identified where the WTA value was lower than the WTP value. Among the included studies, six (17.14%) experiments or subgroups from four studies reported WTA/WTP ratios of less than 1. The lowest ratio, 0.14, was recorded in a study on PMTCT services [36], while the highest in this category was 0.77 from a study on life-delay treatment [29].

Furthermore, two included studies reported WTA/WTP ratios for multiple subgroups [24, 36]. To ensure consistency in the weighting of each article and its respective studies, only the base-case values from these two groups were included in the data statistics and ratio analysis. The ratios reported by these studies fell within the previously mentioned range (0.14–29.19), thus the exclusion of other subgroups did not affect the outcomes of the qualitative analysis.

## Discussion

This study aimed to systematically review the available evidence regarding the disparity between WTA and WTP for health outcomes or healthcare goods and services.

### The current state of WTA and WTP empirical research in the healthcare field

The results of empirical studies may be influenced by the selected methods of value elicitation, specifically concerning WTA and WTP questions. Among the 14 studies addressing both types of WTA and WTP questions, 8 studies revealed a significant discrepancy in response rates, with the majority (6/8, 75%) showing that the response rate for WTA questions was markedly lower than that for WTP questions.

The frequently observed phenomenon of WTA values exceeding WTP values has been consistently reported across numerous similar reviews and systematic reviews. However, this study identified that a certain proportion (6 out of 35, or 17.14%) of empirical studies in the healthcare field reported WTP values surpassing WTA values. This discrepancy may be attributed to the theoretical foundations of the CVM, the low response rates of WTA questions, the nature of public goods, and other influencing factors.

Among the six studies or subgroups reporting higher WTP values than WTA values, four did not disclose the response rates for WTA and WTP questions. In contrast, the two studies that reported response rates indicated that the rates for WTP questions were significantly higher than those for WTA questions [36], with WTP response rates being 1.69 and 4.33 times those of WTA, respectively. This low response rate for WTA questions suggests that respondents may be resistant to providing WTA estimates, potentially leading to underestimation of WTA values.

The measurement of WTA and WTP values relies on individual preference data, which inherently varies among individuals. Moreover, the distinct types of medical and health products and services described in WTA and WTP questions, along with their differing market attributes, can also influence respondents’ preferences. The declarative nature of preferences elicited by the CVM method introduces significant uncertainty into the collected results. Using monetary values to reflect preferences is influenced by various demographic characteristics, with factors such as income, education level, and personal beliefs contributing to the diversity of preferences [20, 23, 37].

Additionally, two empirical studies on the primary prevention of mother-to-child transmission of HIV, conducted by Isah in Nigeria in 2019 and 2021 [33, 36], yielded highly divergent WTA/WTP ratios of 29.19 and 0.14, respectively. This discrepancy may be influenced by factors such as response rates, sample sizes, and the characteristics of the medical and health products examined in these studies. A commonality between both studies is the significantly lower response rate for WTA questions compared to WTP questions. In the 2019 study, for instance, the response rate for WTA questions was only 4.8%, while that for WTP questions was 97.1%. The substantial disparity in sample sizes, along with the potential presence of extreme data and the uncertainty surrounding preferences, likely contributes to the elevated WTA/WTP ratio. Conversely, the significantly lower WTA value in the 2021 study may be attributed to the nature of the product itself. The primary prevention of mother-to-child transmission of HIV represents a public good in Nigeria, where the dynamics of income effects and substitution effects can lead to considerable differences, resulting in a smaller WTA value.

### Reasons for the difference between WTA and WTP

Firstly, the disparity between WTA and WTP question response rates. One potential explanation is that WTP values are generally more influenced by income levels, making it easier for respondents to provide WTP estimates compared to WTA values. However, if low response rates for WTA questions are a widespread phenomenon, this raises questions about the validity of WTA data obtained in empirical studies. A related empirical study found no significant differences between individuals who provided WTA values and those who did not [19]. Nonetheless, qualitative findings suggest that individuals who did not report a WTA value likely possess an internal measure of it. The consistently low adherence to WTA questions, contrasted with high adherence to WTP questions, serves as tangible evidence of differing public perceptions regarding these evaluation methods, thereby reflecting variations in preferences.

Then, the WTA/WTP ratios differ. In various fields of empirical research, it is widely accepted that differences exist between WTP and WTA, although the underlying reasons for these discrepancies remain a topic of debate. A comparison of baseline characteristics among respondents in numerous empirical studies reveals a significant relationship between income levels and the WTA-WTP disparity [20, 21, 27, 33]. This phenomenon can be explained by the income effect in economic theory. Since WTP is constrained by income, whereas compensation claims (WTA) are not, a notable income effect emerges.

The gap between WTA and WTP may widen under certain conditions, such as when the change of concern is substantial, the value of the commodity in question is high, or when the income elasticity of that commodity is significant and increases with income [41, 42]. Specifically, as the value of goods rises, WTP increases until it reaches an income threshold, while WTA can theoretically extend indefinitely. This theoretical pattern is supported by our stratified analysis of country income levels. The median WTA/WTP ratio was highest in low-income countries, while the ratios observed in middle- and high-income countries were relatively similar, suggesting that the valuation gap is more pronounced in lower-income settings. This finding may reflect greater reluctance among individuals in resource-constrained contexts to relinquish healthcare benefits, potentially due to lower economic security, limited access to healthcare, or the heightened perceived value of health interventions.

In addition to the income effect, the substitution effect is a critical factor influencing WTA and WTP in economic theory. While WTP reflects the monetary value of an individual’s willingness to purchase a good, WTA represents the monetary value required to compensate for the rejection of that good, often interpreted as the cost of acquiring a substitute. When fewer substitutes are available, replacing the good becomes more challenging, potentially necessitating higher compensation [41]. Health, being a commodity with few or no substitutes, poses significant difficulty in assigning a monetary value to the loss of a unit of health.

Beyond income levels, other factors contributing to the disparity between WTA and WTP include measurement errors related to technology, individual cognitive preferences, and the resource endowment effect. Almost all empirical studies examining WTA and WTP employ the contingent valuation method, collecting relevant data through questionnaire surveys. The format of WTA and WTP questions can significantly influence the values obtained [43]. For instance, there can be substantial differences between the monetary willingness expressed in open-ended versus closed-ended questionnaires. Furthermore, empirical data are typically summarized using mean values; thus, the presence of extreme data points can skew results and reduce their representativeness.

The resource endowment effect, proposed by psychologists [20], serves as an explanation for observed behaviors regarding WTA and WTP. This effect is linked to loss aversion, where individuals ascribe greater value to goods they own compared to those they do not. Economically, this is expressed as a higher perceived utility for owned goods. Consequently, the compensation required for relinquishing an already owned product often exceeds the monetary value assigned to obtaining a new one, resulting in WTA values that are higher than WTP values. In the healthcare context, the monetary compensation for canceling existing medical services frequently surpasses the amount individuals are willing to pay for new services.

Economic theories explaining the endowment effect emphasize factors such as information costs, uncertainty regarding the value of goods, and the irreversibility of losses [21]. Additionally, the theory encompasses individual cognitive preferences; individuals generally prefer the products or resources they already possess. Variations in education levels and social status can further introduce cognitive biases, leading to differing degrees of disparity between WTA and WTP.

### The “distortion” phenomenon of the cost-effectiveness threshold caused by the disparity between WTA and WTP

**Fig. 2:**
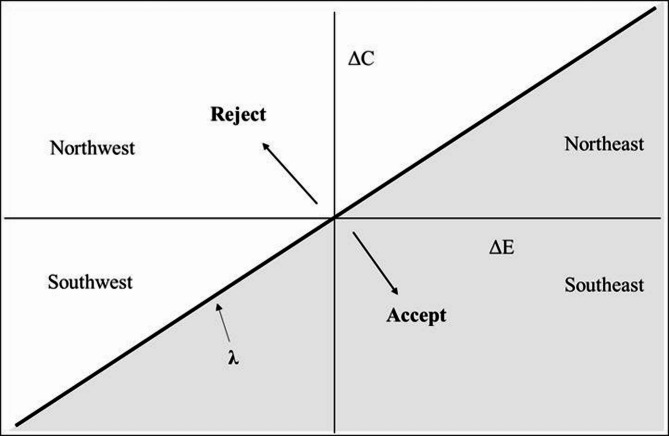
Accept area and reject area in the cost-effectiveness plane without distortion threshold

The cost-effectiveness plane serves as a geometric framework for representing incremental cost (∆C) and incremental effect (∆E) data [7]. In the plane, the horizontal axis denotes ∆E, while the vertical axis indicates ∆C, facilitating clear visualization of the incremental cost-effectiveness ratio (ICER) acceptance or rejection decision rules. The cost-effectiveness plane is divided into four decision regions, with particular attention often given to the upper-right (more effective and more costly) and lower-left (less effective and less costly) regions within pharmacoeconomic research. Points located in the upper-right region signify that a new intervention is both more effective and more costly than the existing intervention. Conversely, points in the lower-left region indicate that the new intervention is less effective and less costly than the old one. In making intervention decisions, it is essential to establish the cost-effectiveness threshold (λ). Most literature defines λ as the slope of a straight line that intersects the origin and spans the upper-right and lower-left region of the cost-effectiveness plane. This implies that the threshold remains constant across both regions, as illustrated in Fig. 2.

Thus, the threshold (λ) serves as a bridge to elucidate the relationship between cost-effectiveness and cost-benefit analysis, providing a mechanism for translating health effects into monetary units [7]. However, it is essential to recognize that this threshold is represented as a straight line, based on the assumption that willingness to pay for health is equivalent to willingness to accept compensation for health loss. In essence, this implies that WTA should equal WTP within the healthcare field.

**Fig. 3:**
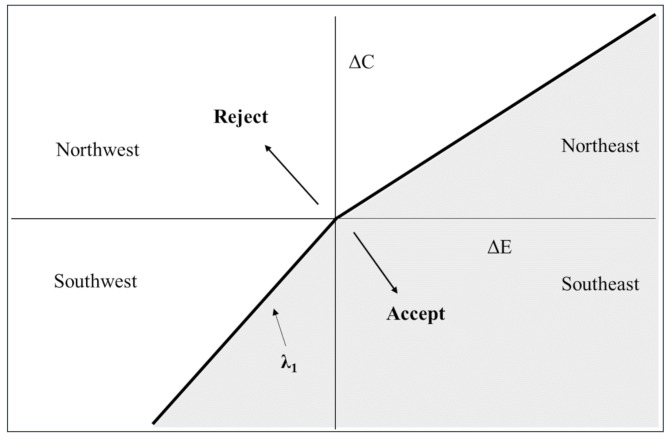
Accept area and reject area in the cost-effectiveness plane with distortion threshold

Empirical evidence, however, confirms that WTA often differs from WTP, which contradicts the notion of a threshold (λ) derived solely from WTP. Consequently, O’Brien argues that cost-effectiveness thresholds in healthcare are distorted [7]. Previous empirical studies in this domain have shown that WTA values are generally higher than WTP values, with the WTA/WTP ratio exhibiting a wide range, reaching as high as 29.19 in the context of this study. Therefore, in the cost-effectiveness plane, the threshold should be represented as a curve that diverges from the origin. In this plane, the slope of the line in the upper-right region corresponds to WTP, while the slope of the line in the lower-left region represents WTA, as illustrated in Fig. 3.

However, in the practical application of pharmacoeconomics, cost-effectiveness analyses comparing intervention programs typically yield ICER values that predominantly fall within the upper-right region [44]. This observation may explain the prevailing emphasis on the WTP threshold. If a new intervention located in the upper-right region, an ICER that remains below the WTP threshold implies that the additional cost per unit of effectiveness (e.g., per Quality-adjusted life year, QALY) is within an acceptable range for policymakers or healthcare payers, suggesting the added value justifies the higher cost [45]. Relatively speaking, if a new intervention demonstrates slightly lower effectiveness compared to an existing one while also incurs reduced costs, yielding an ICER below the threshold-WTA- in the lower-left region, which dramatically suggests that the new intervention may be deemed cost-effective. Since WTA is often higher than WTP, the area under the “kink” threshold is smaller compared to the non-kink threshold, indicating a lower probability of the new intervention being cost-effective [46]. However, healthcare policymakers often focus more on scenarios involving increased effectiveness and costs (the upper-right region), potentially overlooking the implications of cost reductions coupled with marginal decreases in effectiveness. This focus may lead to underappreciation of alternatives that, despite being less effective, offer substantial cost savings and thus warrant consideration in budget-constrained environments.

Pharmacoeconomic evaluation results are frequently utilized by healthcare administrators across various countries as a basis for decision-making. Ignoring the differences between WTP and WTA equates to disregarding the reality that decision thresholds in the upper-right and lower-left region should differ. This oversight could lead to healthcare or insurance decisions based on pharmacoeconomic evidence that are irrational, potentially resulting in societal welfare losses. Therefore, it is imperative to acknowledge and address the disparity between WTA and WTP in pharmacoeconomic evaluations.

### Strengths and limitations

In this study, we employed a systematic approach to estimate the WTA/WTP ratio for health outcome or healthcare goods and services. A key aspect of our analysis involved the collection of response rates from each included study. We examined the differences in response rates between WTA and WTP questions, recognizing their significance in understanding participant engagement and the overall reliability of the data.

Our study, however, also has some limitations. We mainly present a qualitative analysis of the differences between WTA and WTP estimates without incorporating sufficient quantitative analysis. As such, it may not fully capture the comprehensive differences between the two measures. Additionally, we did not include all subgroups from two of the included articles due to an excessive number of reported values, which may introduce a slight reporting bias. However, we believe that neither of these limitations significantly influenced the overall results of the study.

## Conclusion

In conclusion, this systematic review highlights the prevalent disparity between WTA and WTP in health-related valuation studies, with the majority of studies reporting higher WTA values. However, it also reveals that in certain contexts within the medical and health domains, WTP values may exceed WTA. The disparity between WTA and WTP is influenced by various factors, including income and personal preferences, and exhibits significant heterogeneity. Recognizing the differences between WTA and WTP in the medical and health sectors can assist policymakers in making more informed decisions regarding the allocation of medical and health resources. Furthermore, extending the cost-effectiveness threshold to encompass the lower-left region of the cost-effectiveness plane may offer additional options for decision-making in healthcare, thereby optimizing social welfare within resource constraints.

## Supplementary Information

Below is the link to the electronic supplementary material.

Additional file 1: Search strategies

## Data Availability

No datasets were generated or analysed during the current study.

## Abbreviations

CVM: Contingent valuation method
WTP: Willingness-to-pay
WTA: Willingness-to-accept
ICER: Incremental cost-effectiveness ratio
QALY: Quality-adjusted life year
