# Supplementary material for: A systematic review of the willingness-to-accept and willingness-to-pay disparities in empirical studies in the healthcare field
Source: Arch Public Health. 2025 Aug 18;83:212. doi: 10.1186/s13690-025-01699-w (PMC12359863; doi:10.1186/s13690-025-01699-w)
Supplement: Supplementary file 1 — Additional file 1: Search strategies [file 13690_2025_1699_MOESM1_ESM.docx]

**Additional file 1**

**Search strategies**

PubMed

1. wtp[Title/Abstract] OR ‘willingness to pay’[Title/Abstract] OR ‘willingness-to-pay’[Title/Abstract] 9454
2. wta[Title/Abstract] OR ‘willingness to accept’[Title/Abstract] OR (health NEAR/5 forgone[Title/Abstract]) OR (health NEAR/5 foregone[Title/Abstract]) OR (qaly* NEAR/5 forgone[Title/Abstract]) OR (qaly* NEAR/5 foregone[Title/Abstract]) OR (‘quality adjusted life’ NEAR/5 forgone[Title/Abstract]) 1621
3. #1 AND #2 116

Scopus

((TITLE-ABS-KEY (wta OR ( willingness-to-accept ) OR ( health W/5 forgone ) OR (health W/5 foregone) OR (qaly*W/5 forgone) OR (qaly* W/5 foregone) OR (quality-adjusted-life W/5 forgone)) OR TITLE-ABS-KEY ((willingness w/5 accept))) AND (TITLE-ABS-KEY ( wtp OR ( willingness-to-pay )))) AND (TITLE-ABS-KEY( health OR healthcare OR care* OR welfare OR disease* OR practitioner* OR specialist* OR ( familyphysician*) OR medication* OR hospital* OR patient* OR clinical* OR treatment OR therapy OR chemotherapy OR pharmaceut* OR screening OR testing OR vaccin* OR prevention OR preventive ) 227

Embase

1. wtp:ti,ab,kw OR 'willingness to pay' ti,ab,kw OR 'willingness-to-pay':ti,ab,kw 14096
2. wta.ti,ab,kw OR 'wilingness to accept':t,ab,kw OR (health NEAR/5 forgone);ti,ab,kw) OR (heath NEAR/5 foregone):ti,ab,kw) OR (qaly*NEAR/5 forgone):ti,ab,kw) OR ((qaly* NEAR/5 foregone): ti,ab,kw) OR ('quality adjusted life' NEAR/5 forgone):ti,ab,kw) 2374
3. #1 AND #2 141

Web of Science

((TS=(Health OR healthcare OR care* OR welfare OR disease* OR practitioner* OR specialist* OR (family physician* ) OR medication* OR hospital* OR patient* OR clinical* OR treatment OR therapy OR chemotherapy OR pharma* OR screening OR testing OR vaccin* OR prevention OR preventive)) AND TS=("willingness to accept" OR "wta" OR "willingness-to-accept")) AND TS=("willingness to pay" OR "wtp" OR "willingness-to-pay") 243
